# Antiepileptic Properties of Scyllo-Inositol on Pentylenetetrazol-Induced Seizures

**DOI:** 10.3390/ijms24087598

**Published:** 2023-04-20

**Authors:** Karol Wiśniewski, Tomasz Antonowski, Judyta Juranek, Piotr Podlasz, Joanna Wojtkiewicz

**Affiliations:** 1 Students’ Scientific Club of Pathophysiologists, Department of Human Physiology and Pathophysiology, School of Medicine, University of Warmia and Mazury, 10-082 Olsztyn, Poland; 2 Department of Human Physiology and Pathophysiology, School of Medicine, Collegium Medicum, University of Warmia and Mazury, 10-082 Olsztyn, Poland; 3 Department of Pathophysiology, Forensic Veterinary Medicine and Administration, Faculty of Veterinary Medicine, University of Warmia and Mazury, 10-719 Olsztyn, Poland

**Keywords:** scyllo-inositol, seizures, zebrafish, antiepileptic properties, epilepsy

## Abstract

Epilepsy, with about 70 million affected people worldwide, is one of the biggest challenges of medicine today. It is estimated that about one-third of epileptic patients receive inadequate treatment. Inositols have proved effective in many disorders; hence, in the current study, we tested potential antiepileptic properties of scyllo-inositol (SCI)—one of the most common commercially available inositols—in zebrafish larvae with pentylenetetrazol-induced seizures. First, we studied the general effect of SCI on zebrafish motility, and then we tested SCI antiepileptic properties over short (1 h) and long (120 h) exposure protocols. Our results demonstrated that SCI alone does not reduce zebrafish motility regardless of the dose. We also observed that short-term exposure to SCI groups reduced PTZ-treated larva motility compared to controls (*p* < 0.05). In contrast, prolonged exposure did not produce similar results, likely due to the insufficient concentration of SCI given. Our results highlight the potential of SCI use in epilepsy treatment and warrant further clinical studies with inositols as potential seizure-reducing drugs.

## 1. Introduction

Epilepsy is one of the most commonly diagnosed brain disorders worldwide. It is estimated to affect over 70 million people worldwide [1]. Nearly 80% of all epileptic patients live in low- and middle-income countries, and of these over 75% remain untreated, constituting a significant treatment gap in these countries [2]. Although there are many commercially available treatment options, about 1/3 of all epileptic patients do not respond positively to any of the available medications, making the treatment ineffective and leading to recurrent seizures in these patients [3]. Inadequately treated epilepsy reduces quality of life, affecting both patients and their caretakers and burdening the healthcare system.

Scyllo-ins (SCI) belongs to the Inositol (ins) family of carbocyclic sugars, implicated in membrane biogenesis, antioxidation, signal transduction, and phosphate storage [4,5]. There are currently nine known forms of ins: SCI, myo-inositol (MI), neo-inositol, epi-inositol, muco-inositol, cis-inositol, allo-inositol, L-chiro-inositol, and D-chiro-inositol [6] They are frequently used in conjunction with other medications to treat a variety of conditions, including diabetes, polycystic ovary syndrome, metabolic syndrome, psoriasis, depression, panic attacks, obsessive-compulsive disorder, etc. [5,7,8,9,10]. SCI supplementation has been shown to be useful in treating Alzheimer’s [11] and Huntington’s disease [12], and there is evidence that hepatic encephalopathy causes SCI depletion [13]. Additionally, SCI is being investigated as a potential treatment for dementia in Down syndrome patients [14] and as a marker for tumor treatment surveillance in brain proton magnetic resonance spectroscopy (MR spectroscopy) [15]. This evidence highlights the potential role of SCI in the control of nervous system activity.

The zebrafish is a helpful model in various neurological diseases, allowing researchers to conduct detailed genetic, embryological, developmental and behavioral analyses. Zebrafish larvae exhibit measurable neurobehavioral traits, such as those related to human sleep, learning, addiction, and other behaviors [16,17,18]. The organization of the zebrafish brain is similar to that of other vertebrates [19], and central neurotransmitter systems (cholinergic, noradrenergic and dopaminergic) are not only present but also have been mapped throughout the entire brain [20]. Furthermore, thanks to their translucent bodies and small size (approximately 4 mm at four dpf or days post-fertilization), it is easy to conduct quantitative and qualitative studies, making zebrafish one of the most useful model organisms.

Pentylenetetrazol (PTZ) is a GABA (gamma-aminobutyric acid)—a receptor antagonist [21]—and a well-established convulsant drug, formerly used as a cardio-respiratory stimulant [22]. Recently, it has become the first drug of choice in epilepsy studies, used to induce seizures in laboratory animals [23,24,25]. PTZ triggers epileptic seizures by blocking the inhibitory action of GABA-A and causing neuronal hyperexcitation [26,27], leading to dose-dependent seizures.

In this study we aimed to determine the antiepileptic properties of SCI in zebrafish larvae with PTZ-induced seizures.

## 2. Results

### 2.1. SCI Effect on Zebrafish Larva Motility

**Figure 1:**
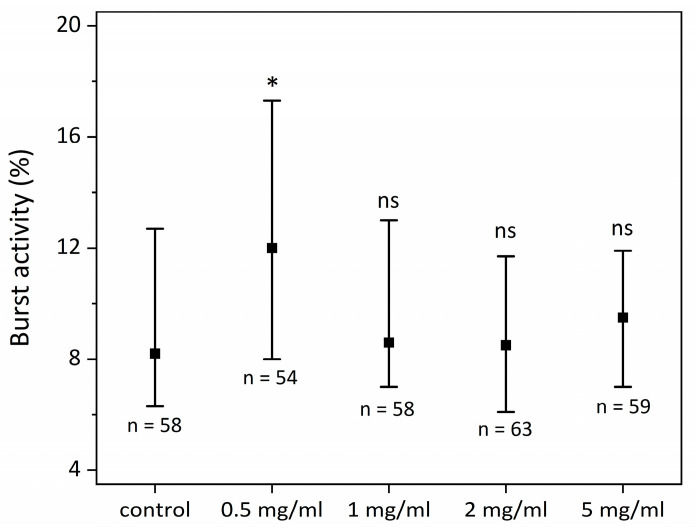
Comparison of zebrafish larva motility after SCI exposure, * *p* < 0.01 compared to the control group, ns—not significant.

First, we studied the effect of SCI exposure on zebrafish motility in control, non-PZT-treated zebrafish embryos. The results are shown in Figure 1. Our data demonstrated that the 0.5 mg/mL group had significantly higher motility than all other groups. No statistically significant differences were observed within and between groups.

### 2.2. Antiepileptic Properties of SCI on Zebrafish Larvae—Short-Term Exposure to SCI

**Figure 2:**
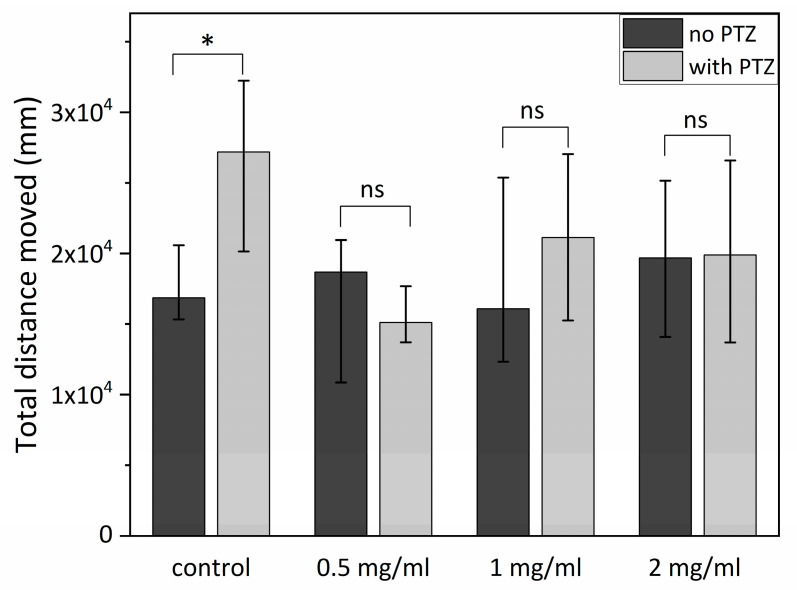
Comparison between matching groups exposed and not exposed to PTZ in short-term exposure protocol; * *p* < 0.05; ns—not significant.

**Figure 3:**
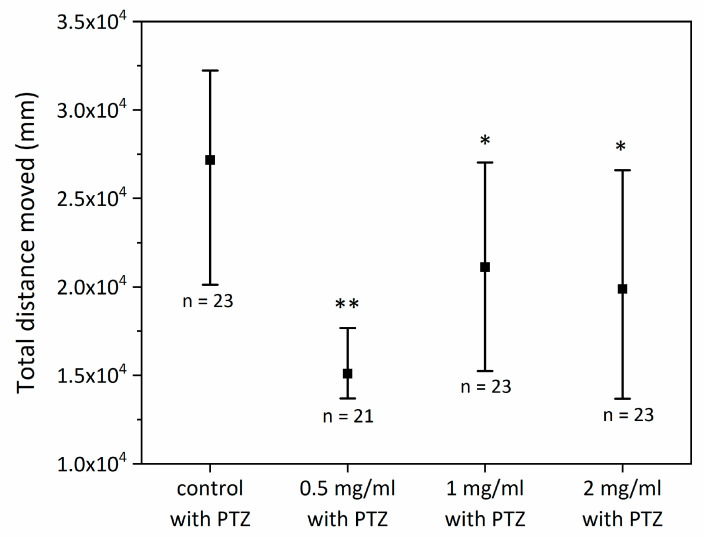
Comparison of different groups exposed to PTZ in short-term exposure protocol. * *p* < 0.05 compared to control group; ** *p* < 0.0001 compared to control.

First, the comparison between healthy and PTZ-stimulated groups was performed. The results of this analysis are presented in Figure 2. A statistically significant difference was observed between control groups (*p* < 0.01). However, no difference was observed (*p* = ns) between healthy and PTZ-stimulated SCI groups. Moreover, a significant difference was observed after PTZ stimulation between the control and each SCI group (0.5 mg/mL, 1 mg/mL, 2 mg/mL). Furthermore, the 0.5 mg/mL group had a significantly better response following PTZ stimulation (less motility) than the 1 mg/mL group or the 2 mg/mL group. Between the 1 mg/mL group and the 2 mg/mL group, no significant difference was observed. The results are presented in Figure 3.

### 2.3. Antiepileptic Properties of SCI on Zebrafish Larvae—Long-Term Exposure to SCI 

**Figure 4:**
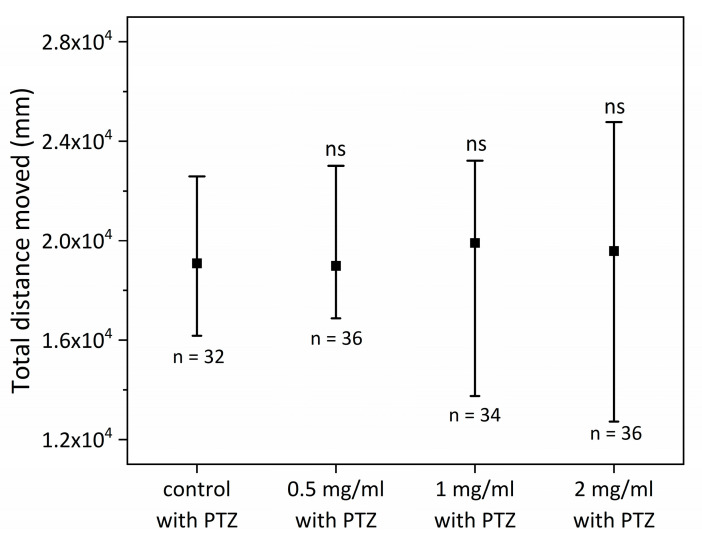
Comparison of different groups exposed to PTZ in long-term exposure protocol; ns—not significant.

Similarly, in the long-term exposure protocol, the comparison between healthy and PTZ-stimulated groups exposed to different concentrations of SCI was performed. No significant differences between either the control group and SCI groups or between SCI groups were noticed (Figure 4).

## 3. Discussion

First, we investigated whether zebrafish larval motility was negatively impacted by SCI exposure by comparing the larval motility between the control and SCI. Our findings demonstrated that SCI not only decreases zebrafish motility but also increases it—at least at concentrations of 0.5 mg/mL of SCI. The discovery is significant as it supports the prospective use of SCI as an antiepileptic drug. Zebrafish larva motility may increase in SCI groups either due to SCI directly connecting to GABA-A receptors or indirectly through SCI metabolites, most likely through binding to excitatory receptors in the brain [28].

Furthermore, to investigate the potential antiepileptic properties of SCI, we used seizure-inducing PTZ to increase zebrafish larva motility. The total traveled distance of each larva was tracked and quantified. In the short-term exposure protocol, the results showed that each of the SCI groups (0.5 mg/mL, 1 mg/mL, 2 mg/mL) exposed to PTZ had a statistically significant shorter total traveled distance compared to the control group exposed to PTZ but not receiving SCI treatment, highlighting the potential of SCI in seizure treatment. Furthermore, the comparison between healthy (control) and PTZ-exposed groups revealed statistically significant differences only between controls groups but not between SCI groups. We observed that SCI groups exposed to PTZ had a similar level of motility compared to their counterparts not exposed to PTZ, albeit a significantly lower level compared to the control group exposed to PTZ but not exposed to SCI. This observation indicates that SCI has beneficial effects, reducing motility in zebrafish larvae exposed to PTZ. Furthermore, in the same experiment, the 0.5 mg/mL group displayed significantly lower motility compared to other SCI groups (1 mg/mL, 2 mg/mL), highlighting the potential of SCI in epilepsy treatment. It is noteworthy, though, that among groups exposed to different SCI concentrations, the best results were observed at the lowest SCI concentration, i.e., 0.5 mg/mL SCI group, diminishing with higher doses likely due to SCI saturation [29].

Finally, we investigated whether long-term SCI exposure may reduce PTZ-induced seizures in zebrafish larvae more effectively than short-term exposure. Contrary to the short-term exposure, no significant difference between groups exposed to PTZ was noticed. However, the largest offsets of measured values were observed in the 1 mg/mL and 2 mg/mL groups, suggesting that certain zebrafish embryos may respond to SCI more favorably than others. Additionally, it is likely that the protocol’s SCI dosages were insufficient to produce therapeutic effects, hence no modifications in motility were noted. The other possible explanation is the fact that the seizure induction in this part of the experiment was insufficient (total distance moved <2.0 × 10^4^ mm versus >2.75 × 10^4^ mm in the short protocol) which may also be the reason for no SCI antiepileptic effect in long exposure protocol. Further studies are required to fully understand the underlying cause of this phenomenon.

Nozadze et al. (2010) evaluated the antiepileptic effects of SCI or MI administration in a rat model of epilepsy, in which seizures were also induced by PTZ. PTZ administration took place 30 min after SCI or MI administration. In agreement with our short-term exposure protocol findings, their study revealed that the MI and SCI treatments dramatically decreased the severity and length of the seizures while increasing the latent period [30].

In 1996, M. H. Richards and colleagues looked into the efficacy of inositols (myo-, sci-, epi-L-chiro) in reversing carbachol/lithium-stimulated CMP-PA buildup. According to the study’s findings, both MI and epi-inositol (EI) suppressed the stimulation triggered by carbachol–lithium in a concentration-dependent manner (10 mmol of MI caused full inhibition, 30 mmol of EI caused 83.4 percent inhibition). In contrast, SCI, at a concentration of 25 mmol, reduced the reaction by 17.5 percent. SCI reduced the response by 27.3 percent (again, at a dosage of 25 mmol/L) in the second cell model that was evaluated, and the inhibition was not dose-dependent [31].

In our experiments, SCI concentrations were lower (25 mmol = 4.5 mg/mL) than those described in the study, likely explaining the lack of any significant differences observed in the long-term exposure protocol. Further studies using higher concentrations of SCI are needed to fully investigate SCI potential as an antiepileptic molecule.

There are a few potential molecular explanations for MI’s antiepileptic effects, but it is unclear how this translates into the potential antiepileptic activity of SCI. Currently, it is understood that SCI and MI are similar in terms of both structure and mode of action [6]. Some of the oral MI supplement is converted in the body to SCI after ingestion [32]. Additionally, it appears that MI and SCI are both taken up into cells by the same active transporters [33], and that brain tissue contains the highest concentration of both of them (100-fold greater than in the surrounding tissues) [11].

There is evidence that in vitro MI inhibits 3H muscimol, a specific ligand of the GABA binding site at the GABAA receptor, and stimulates 3H MK-801, a specific ligand of activated NMDA-type glutamate receptors, in rat brain membranes [34], modulating GABA-A and NMDA receptors and thereby exerting its anticonvulsant action. The osmotic activity of MI may also be a contributing factor to its antiepileptic effects. A massive influx of Na^+^, Ca^2+^, Cl, and water occurs at the increased rates of neuronal activity, producing cellular swelling. High osmolyte concentrations build up in cells to balance the influx and minimize swelling while preserving function of all essential enzymes [35,36,37]. The antiepileptic effects of SCI might likely be carried out by the same molecular processes; however, more mechanistic studies are required to uncover these processes.

## 4. Methods and Materials

The wild zebrafish strain (Tu-Tubigen) was set for spawning in spawning containers. Eggs in stadium 0.25–1 h post-fertilization were collected and rinsed in E3 solution (5 mM NaCl, 0.17 mM KCL, 0.33 mM CaCl_2__H_2_O, 0.33 mM MgCl_2__6H_2_O, and pH 7.2), and selected for further processing. 

### 4.1. SCI Effect on Zebrafish Larvae Activity

Selected eggs were incubated in E3 solution for 24 h at 28 °C. Next, Petri dishes were prepared for five tested groups, 32 individuals per well. Experimental groups were as follows: 0 mg/mL SCI, 0.5 mg/mL SCI, 1 mg/mL SCI, 2 mg/mL SCI, and 5 mg/mL SCI diluted in E3 solution. Prepared culture plates were incubated for 1 h at 28 °C, followed by quantitative analysis of zebrafish larva motility using DanioScope v 1.2.206 ((Noldus, Wageningen, The Netherlands) [38]—burst activity%).

### 4.2. Antiepileptic Properties of SCI on Zebrafish Larvae—Short-Term Exposure to SCI

Selected eggs were incubated in E3 solution for 24 h at 28 °C. At 24 hpf (hours post-fertilization), 48 hpf, 72 hpf, and 96 hpf solutions were changed. At 120 hpf, stadium Petri dishes were prepared for four tested groups. The following tested groups were prepared: control (0 mg/mL SCI), 0.5 mg/mL SCI, 1 mg/mL SCI, and 2 mg/mL SCI (60 individuals per group) and incubated for 1 h at 28 °C. Following the incubation, 48 individuals were randomly selected and transported to 48 well plates (one individual per well). A volume of 200 mL of E3 solution was added to each well. Five minutes before analysis, 36 individuals from each tested group were incubated with 10 mmol PTZ to mimic epileptic seizures. The remaining specimens from each tested group were used as controls (*n* = 12, no PTZ). 

### 4.3. Antiepileptic Properties of SCI on Zebrafish Larvae—Long Exposure to SCI

Selected eggs were incubated in E3 solution for 24 h at 28 °C. At the 24 hpf stadium, zebrafish larvae were randomly assigned to each of four tested groups (60 individuals per group): 0 mg/mL SCI, 0.5 mg/mL SCI, 1 mg/mL SCI, and 2 mg/mL SCI. At 48 hpf, 72 hpf, 96 hpf, and 120 hpf solutions were changed. At the 120 hpf stadium, 48 individuals were randomly selected and transported to 48 well plates (one individual per well). A volume of 200 mL of E3 solution was added to each well. Five minutes before analysis, 36 individuals from each test were incubated with 10 mmol PTZ. The remaining specimens from each tested group were used as controls (*n* = 12, no PTZ).

### 4.4. Statistical Analysis

All measurements were performed using DanioVision system with Ethovision XT v.15 software (Noldus, Wageningen, The Netherlands) [34]. The observation time for each larva was 10 min in both exposure protocols. The used parameter was the total distance moved (mm). After collecting data, Dixon’s Q test was conducted in Excel (Microsoft Corporation, Redmond, WA, USA), followed by statistical analysis in Statistica 13.3 (TIBCO Software Inc., Palo Alto, CA, USA). The Mann–Whitney U test was used to calculate the statistical significance. The *p* < 0.05 was considered statistically significant. Figures were prepared using OriginPro 2020 (OriginLab Corporation, Northampton, MA, USA).

## 5. Conclusions

One of modern medicine’s priorities is the treatment of epilepsy. Despite the availability of a wide variety of antiepileptic medications, it is thought that even one-third of the 70 million affected individuals do not receive proper care. Utilizing endogenous chemicals, such as SCI, may alter the situation. Identifying potential antiepileptic properties of SCI on PTZ-induced seizures in zebrafish larvae was the goal of this study. Our study’s results demonstrate that SCI doses up to 5 mg/mL do not appear to have an effect on the zebrafish larvae’s ability to move, suggesting that they might be employed in additional preclinical investigations without risk. As a result of short-term exposure, our findings also indicated that SCIs have some antiepileptic qualities. Unfortunately, the long-term SCI exposure protocol did not produce any discernible effects, likely due to the insufficient SCI concentration used in our study. Nevertheless, SCI should be considered as a potential antiepileptic medication in future studies of epilepsy therapy. 

## Data Availability

Data available on request from the authors.
